# Negative Impacts of School Class Segregation on Migrant Children’s Education Expectations and the Associated Mitigating Mechanism

**DOI:** 10.3390/ijerph192214882

**Published:** 2022-11-12

**Authors:** Cixian Lv, Xiaotong Zhi, Jingjing Xu, Peijin Yang, Xinghua Wang

**Affiliations:** Normal College, Qingdao University, Qingdao 266071, China

**Keywords:** migrant children, college entrance examination for migrant children, policy identification, school class segregation, educational expectation

## Abstract

This study thoroughly analyzes the impacts of school class segregation on the four dimensions of educational expectations of migrant children, and verifies the moderating effects of migrant children’s identification with the college entrance examination policy on the relationship between the two. A total of 1770 questionnaires were collected for this study. Through multiple regression analysis and moderating effect tests on the data, this study reveals that school class segregation has a significant negative impact on the educational expectations of migrant children; the migrant children’s identification with the college entrance examination policy also partially moderates the impacts of school class segregation on the academic achievement expectations and interpersonal expectations of migrant children. Informed by these results, this study proposes the following three mechanisms that can be used to mitigate the negative impacts of school class segregation on migrant children’s educational expectations: (a) an institutional mechanism involving the “unified urban–rural household registration”; (b) a cultural mechanism involving “promoting learning through examinations”; (c) a compensation mechanism involving the “principle of justice”. This paper provides a Chinese perspective on the issue of school class segregation by offering a policy reference for the improvement of the college entrance examination policy for migrant children and the reform of the household registration system.

## 1. Introduction

In China, the household registration (hukou) system categorizes people into rural and urban residents to control rural-to-urban migration. After the socioeconomic reform in the late 1970s, rural residents were allowed to migrate to cities and live in there to meet labor demands. In this process, a special group of migrant children emerged, who followed their parents from the rural areas to the cities [1]. However, there are some barriers preventing them from taking the college entrance examination in the places of inflow. It was not until the reform of the college entrance examination policy for migrant children that the problem was addressed. Kerckhoff believed that education, particularly higher education, helps individuals acquire and improve their social status, and is a key force for the stratification and mobility of modern society [2]. According to China’s National Education Development Statistics Bulletin in 2020, migrant children who had moved to cities accounted for 14.2696 million students in compulsory education, accounting for approximately 9.1% of the general student population [3]. Among them, there were 10.3486 million primary school children and 3.9488 million junior school children. Therefore, China has been attaching great importance to the education of migrant children. They have been included in the national scheme of “poverty alleviation by improving education”, for the purpose of blocking the “intergenerational transmission of poverty”. With little doubt, the research relating to the education of migrant children is of great practical significance.

Generally, either educational expectations or educational attainments can be representative of the student’s educational background to a certain extent. The educational expectation is a subjective social psychology variable that can directly or indirectly explain the student’s educational attainment and status acquisition [4]. School class composition is one of the important factors affecting the student’s educational expectation or educational attainment. Coleman first emphasized in 1966 the importance of school class composition to the academic achievement of specific groups [5]. Numerous empirical studies have proven that the higher the school class composition, the greater the students’ educational expectations [6,7,8]. Furthermore, significant others (such as schoolteachers and learning partners) also have an important impact on academic achievements and educational expectations in social interactions [9,10]. In general, existing studies have confirmed a stable and robust relationship between school class composition and students’ educational expectations.

In China, because of the household registration system, migrant children are not eligible to enjoy the various benefits provided by the government to urban residents [11]. They usually attend marginalized public schools or schools specialized for migrant children. These schools are not as good as other schools in terms of class composition and quality of teaching. As such, this phenomenon begs questions: does this phenomenon of class segregation in China affect the educational expectations of migrant children? With the advancement of the reform of the household registration system and the improvement of the college entrance examination policy in China, the issue of how to mitigate the negative impacts of school class segregation on the educational expectations of migrant children has become an important research direction.

To address these challenges, this study employed stratified sampling to randomly select school-age migrant children from 11 regions where migrant children are concentrated and have implemented the college entrance examination policy specialized for migrant children. We collected 1770 valid questionnaires. Through multiple regression analysis and moderating effect tests, this study analyzed the negative impacts of school class segregation on the educational expectations of migrant children and the moderating effects of policy identification on the relationship between the two.

## 2. Theoretical Foundations and Research Hypotheses

### 2.1. School Class Segregation: Its Connotations and Causes

Segregation is the degree to which two or more groups are separated from each other [12]. School class segregation means that groups of students from different classes gather at different schools, resulting in insufficient heterogeneity in the school class composition. Blau argues that “heterogeneity” is a concept as opposed to “segregation” [13]. The lower the degree of segregation, the higher the heterogeneity. Heterogeneity weakens the negative impact of segregation on intergroup communication, while segregation also weakens the positive impact of heterogeneity on intergroup communication.

In Western countries, students of different races tend to gather at schools of different races, and there is almost no communication among these schools. This phenomenon is called school racial segregation [14]. Furthermore, studies in Western contexts show that due to factors such as the quality of school teaching, housing prices in school districts, and the quality of public services, people of different socioeconomic positions experience obvious school class segregation when choosing schools [15,16]. Higher-class families tend to choose to live with groups close to their class. Such behaviors have contributed to the situation that education and other public services in these small settlements are superior to those where lower-class families live. Low-class families usually lack access to high-quality school districts because they cannot afford expensive school district housing. In line with the analyses above, it can be seen that the phenomenon of school class segregation in Western countries stems from the self-selection and interaction of different class groups at the micro-level.

In China, the issue of school class segregation has certain characteristics, mainly due to the macro-level education policies and the differentiation in the quality of school education under policy changes. The issue of school class segregation in China results from unequal resources distribution in primary and secondary schools. In 1962 when China was newly established, public schools were divided into key schools and non-key schools, concentrating superior resources on key schools. This system has caused a significant difference in the quality of operation between key schools and non-key schools. In 2006, the Compulsory Education Law of the People’s Republic of China terminated the key school system, replacing the “junior high school entrance examination” with “enrolling in nearby schools”, and replacing “key schools” with “demonstration schools” [17]. However, the original issue of differentiation in the quality of education between key schools and non-key schools still exists. The essence of the system of “enrolling in nearby schools” is to distribute educational resources based on the division of places of residence, but there is still a certain degree of difference in the quality of operation among schools in different places of residence. Some parents would enable their entitled children to attend higher-quality schools by “paying to enroll their children in better schools” or purchasing high-quality school district housing. To a large extent, this has caused the tendency for children from higher-class families to concentrate on high-quality schools, while the children from lower-class families without financial means have to choose low-quality schools.

As the policy progressed, some public schools replaced the schools specialized for migrant children and have become the main channel for migrant children to receive education [18]. However, these public schools are usually the schools with a low quality of education and poor operating conditions and are located in the rural–urban fringe zone. The phenomenon of “class stratification” similar to “White Flight” [19] also emerged in these public schools. In other words, local high-class children constantly choose to leave these schools. As a result, even if these migrant children are in such public schools, the “class heterogeneity” of these schools has gradually declined.

### 2.2. Relationship between School Class Segregation and Educational Expectations

Coleman once pointed out that there were significant differences in academic achievements among students from different classes, and that student class composition in a school significantly affected the academic achievement of the students [5]. Following the Coleman report, based on the cohort effect in the social interaction theory, extensive studies have explained how school class segregation affects students’ academic achievement and educational attainment, and have argued that school class segregation directly affects students’ educational expectations [8,20]. In the process of interacting with peer groups, students gradually establish their educational expectations, especially those uncertain about their future paths [21,22]. School class composition determines what type of peer group an individual will interact with. If disadvantaged groups are in a school with a larger percentage of advantageous class students, this would be more conducive to their educational expectations and academic achievements, because advantageous class students usually have better family social capital, better noncognitive habits, and higher educational expectations. On the contrary, if disadvantaged groups are in a school where disadvantaged class students are concentrated, this would have a negative impact on their educational expectations and academic achievements, because such peer groups are usually adverse to learning and even anti-school [23]. It can be seen that school class composition affects the atmosphere of the school and the cohort subculture, which would undoubtedly have both tangible and intangible profound impacts on students.

Currently, the schools that migrant children attend are usually the marginalized public schools or the schools for the children of low-class migrant workers [24]. School types affect the cultural production of migrant children. In other words, "self-abandonment culture" prevails in the marginalized public schools, and there is a certain “anti-school culture” in the schools for migrant children [25]. Even when migrant children attend public schools, they are still in a “subculture” state where they identify with school education but lack confident and positive self-orientation [26]. The outstanding students among them tend to find it difficult to form good educational expectations because of the situation of “class segregation” and the restrictions on the entrance examinations for senior middle schools and colleges. However, if the heterogeneity of school classes is increased, students in the disadvantaged social class can achieve better educational achievement and educational expectations in the school with higher social classes on average [27].

However, researchers have not reached a consensus regarding the classification of educational expectations. Some scholars believe that educational expectation is one-dimensional and mainly emphasizes the expectation for future academic qualifications [28,29]. However, other scholars believe that educational expectations should have two or more dimensions. For example, Rodman and Voydanoff believed that educational expectations include academic achievement expectations and future career expectations [30]; Nikitina and Furuoka thought that educational expectations should be varied and diverse and students’ expectations of university education could be separated into three categories, such as “Life Skills”, “Subject Matter (Hard Skills)”, and “Soft Skills” [31]. This paper considers that migrant children are different from urban students of the same age, and their living conditions are more influenced by the social environments and policy environments. In a complex environment, they wish to be on par with urban counterparts in terms of moral performance and to be better integrated into the cohort. Therefore, in addition to common academic achievement expectations and social achievement expectations, this study proposes that the educational expectations of migrant children also include moral performance expectations and interpersonal expectations.

Based on the above research, this paper proposes the following hypotheses about the relationship between school class segregation and the educational expectations of migrant children: 

School class segregation has a significant impact on migrant children’s academic achievement expectations (H1a), their moral performance expectations (H1b), their interpersonal expectations (H1c), and their social achievement expectations (H1d).

### 2.3. Relationship between the College Entrance Examination Policy for Migrant Children and Their Educational Expectations

With the introduction of national policies supporting compulsory education, the issue of migrant children falling short of receiving fundamental education in the place of inflow has largely been solved. However, the limited access to college entrance examinations in the place of inflow still existed and was finally addressed by the implementation of the college entrance examination policy specialized for migrant children in the year of 2012. 

Prior to the application of the college entrance examination policy for migrant children, scholars studied the educational expectations of migrant children from the perspective of the household registration system. The household registration system is accompanied by indelible identity labels and discrimination, which affect students’ access to social support in schools [32]. Students with different household registration usually have different educational expectations, and nonlocally registered students who cannot participate in local matriculation examinations in the place of inflow have significantly lower expectations for years of schooling [33,34]. It can be seen from this that the household-registration-based difficulties of migrant children in continuing their studies in the localities have blocked the establishment of their good educational expectations. Drawing on the “theory of culture and ecology” [35], school class segregation and marginalized living environment are the social foundation for forming subcultures such as “self-abandonment culture” and “anti-school culture” of migrant children. In order to obtain an in-depth understanding of the school education of disadvantaged groups, we should take into account both institutional factors and the subjectivity of disadvantaged groups. In particular, we need to pay attention to the adapting behaviors of disadvantaged groups when they face institutional barriers.

The success of policy implementation hinges on the identification and support of the policy objects [36]. Whether the college entrance examination policy for migrant children can actually work also largely depends on the degree to which the policy objects identify with it. Based on this and from the perspective of essence and quantification of policy implementation, we used the psychological variable of “migrant children’s identification with the college entrance examination policy” to study the impacts of the college entrance examination policy for migrant children on the educational expectations of migrant children and on the relationship between school class segregation and educational expectations. Based on the above analysis, this paper proposes the following hypotheses:

Migrant children’s identification with the policy has a significant impact on their academic achievement expectations (H2a), moral performance expectations (H2b), interpersonal expectations (H2c), and social achievement expectations (H2d).

Migrant children’s policy identification has a moderating effect on the relationships between school class segregation and their academic achievement expectations (H3a), moral performance expectations (H3b), interpersonal expectations (H3c), and social achievement expectations (H3d).

## 3. Research Design and Variable Measurement

### 3.1. Research context

Among the 10 provinces that first implemented the college entrance examination policy for migrant children, 11 regions with a high concentration of migrant children as targeted regions were selected, including Hangzhou City in Zhejiang Province, Ningbo City in Zhejiang Province, Fuzhou City in Fujian Province, Nanjing City in Jiangsu Province, Qingdao City in Shandong Province, Hefei City in Anhui Province, Nanchang City in Jiangxi Province, Wuhan City in Hubei Province, Changsha City in Hunan Province, Shijiazhuang City in Hebei Province, and Zhengzhou City in Henan Province. The stratified sampling method was employed, through which the students in grades 8 to 11 (aged between 13 and 16 years old) from 15 schools in these 11 regions were selected to join the study. They were generally from rural areas with low socioeconomic status. They come to the cities to study because of their parents’ work, which, in many cases, are low-end jobs. Therefore, the migration of the participants in this study happened within the border of the same country. In this sense, they are different from immigrants in some Western countries. Through on-site screening of the questionnaires and the tabulation removal method, the research team collected a total of 1770 valid questionnaires, with a valid collection rate of 82.3%.

The descriptive statistics of the questionnaires are shown in Table 1.

As shown in Table 1, in response to “Duration of residence”, 88.6% of the respondents reported “3–4 years or more” in the inflow cities. Most of the migrant children lived and studied in the place of inflow for a long time. Thus, the children of migrant workers are different from the “college entrance examination migrants” in the general sense. The “college entrance examination migrants” and the college entrance examination specialized for migrant children are two closely related issues under the household registration system. The “college entrance examination migrants” refers to the group of students who illegally move their school enrollment status to a region where the college entrance examination is simpler, but actually attend school in their birth origins, for the purpose of increasing the success rates of entering good universities. In response to “Type of household mobility”, 79.4% of the survey respondents were students who flowed from rural areas to towns. Among migrant children, 75.1% of them attended local public schools. The age distribution of the survey respondents ranged from the 8th to 11th grade. Among them, the students of the 8th grade accounted for 30.1%, the students of the 9th grade made up 34.9%, those of the 10th grade 17.6%, and those of the 11th grade 17.4%.

### 3.2. Variable Definitions and Measurement

Likert scales were used in this study, including the “Scale of Migrant Children’s Identification with the College Entrance Examination Policy”, the “Scale of Migrant children’s Education Expectations”, and the “Scale of School Class Segregation”. All scales were rated on 5 levels, with the intensity increasing from level 1 to level 5.

#### 3.2.1. Migrant Children’s Identification with the College Entrance Examination Policy

“Identification” refers to an intrinsic recognition of a contextual symbol by an individual through continuous evaluation in the course of social activity [37]. Similarly, “migrant children’s identification with the college entrance examination policy” refers to migrant children’s psychological recognition, acceptance, and support for the college entrance examination policy. Parsons’ system theory [38] proposes a method for measuring policy identification and dividing its dimensions. However, there is no mature scale of policy identification in existing studies. Regarding Parsons’ system theory, this study developed the scale for migrant children’s identification with the college entrance examination policy from three dimensions, including policy awareness, policy emotion, and policy evaluation. Through small sample forecasting and data reliability tests, we revised the items and finally created a 9-item scale for measuring migrant children’s identification with the college entrance examination policy. The Cronbach’s alpha coefficient was 0.965, and the CITC value for each measurement item was greater than 0.5. The validity was tested by the confirmatory factor analysis. The composite reliability was 0.967 and the construct validity was 0.768. The first-order confirmatory factor model fitted the formal sample data.

#### 3.2.2. Educational Expectations of Migrant Children

“Educational expectation” refers to an individual’s expectation for future educational achievement (such as the highest level of educational achievement) and realistic beliefs or judgments about future educational achievement [39]. Educational expectations of migrant children are formed in the process of getting along with important others such as parents and teachers [40]. In this process, migrant children would gradually establish their expectations and goals, and gradually internalize them into their daily behavior patterns. Through literature review [28,30,31], this paper measured the educational expectations of migrant children from four dimensions including academic achievement expectations, moral performance expectations, interpersonal expectations, and social achievement expectations. The questionnaire of educational expectations consists of a total of 16 items, with a Cronbach’s alpha coefficient of 0.968. It includes 4 items on academic achievement expectations with a Cronbach’s alpha coefficient of 0.93, 4 items on moral performance expectations with a Cronbach’s alpha coefficient of 0.872, 4 items on interpersonal expectations with a Cronbach’s alpha coefficient of 0.901, and 4 items on social achievement expectations with a Cronbach’s alpha coefficient of 0.867. The CITC value for each measurement item was greater than 0.5. We constructed a confirmatory factor model for educational expectations. The composite reliabilities of the four dimensions were 0.933, 0.873, 0.912, and 0.879, respectively. The construction validities were 0.779, 0.635, 0.723, and 0.647, respectively, and the second-order four-factor confirmatory factor model basically fitted the formal sample data.

#### 3.2.3. School Class Segregation

School class segregation is the core independent variable of this paper. This study mainly examined the impact of migrant children’s school class segregation on their educational expectations and also investigated the moderating effects of migrant children’s identification with the college entrance examination policy on the relationship between the two. We consulted the “five dimensions of segregation” [41], “three variables composition of school class” [8], and “household registration segregation coefficient” [42], which were well accepted in previous relevant studies, and introduced “school class composition” to explain the state of school class segregation. The scale of school class segregation consists of 5 measurement items, with a Cronbach’s alpha coefficient of 0.852. We constructed a confirmatory factor model for school class segregation. The composite reliability was 0.881, the construct validity was 0.599, and the first-order confirmatory factor model fitted the formal sample data.

### 3.3. Data Analysis

Through correlation analysis, this paper initially verified the correlation between two variables (school class segregation, and migrant children’s identification with the college entrance examination policy) and the educational expectations of migrant children in various dimensions. Then, this paper employed regression analysis to test moderating effects, focusing on the moderating effects of migrant children’s identification with the college entrance examination policy on the relationship between school class segregation and educational expectations in various dimensions. As the educational expectations of migrant children vary to different degrees in terms of demographics, in the regression analysis, we included these demographic variables as control variables in the regression model. In summary, by employing the above methods, this paper analyzed the negative effects of school class segregation on the educational expectations of migrant children.

## 4. Research Conclusions

### 4.1. Correlation Analysis

The correlation analysis results of school class segregation, migrant children’s identification with the college entrance examination policy, and educational expectations are shown in Table 2. Among them, there was a significant negative correlation between school class segregation and the education expectations of migrant children in various dimensions, and there was also a significant positive correlation between migrant children’s identification with the college entrance examination policy and the educational expectations of migrant children in various dimensions.

### 4.2. Regression Analysis

In order to ensure a scientific and reasonable explanation with multilinear regression models, we need to investigate the multiple collinearities, sequence correlation, and heteroskedasticity of the model, etc. The multicollinearity test was tested through tolerance and the variance inflation factor (VIF). In this study, the VIF of each variable in each model was between 0 and 3, which showed that there was no multicollinearity problem. As this study did not involve the comparison of multi-period sample values, and the DW value of each regression model was close to 2, there was no sequence correlation problem in each model. The scatterplot of the residual terms was used to determine the heteroskedasticity problem. In this study, the scatter plot of each regression model was disordered, and the heteroscedasticity problem usually appeared in time series data. As such, the problem of heteroscedasticity was minimal in this study.

#### 4.2.1. Testing of the Moderating Effects of Policy Identification on the Relationship between School Class Segregation and Academic Achievement Expectations

Table 3 shows the results of testing the moderating effects of migrant children’s identification with the college entrance examination policy on the relationship between school class segregation and academic achievement expectations. The explanatory powers of the models of four orders were 0.367, 0.555, 0.761, and 0.767, respectively, and all variables could explain 76.7% of the variance of the dependent variable. It was found through the F test that the explanatory powers were statistically significant.

Based on Model 1, Model 2 added an independent variable (school class segregation). This variable had a significant negative predictive effect on academic achievement expectations (*β* = −0.789, *p* < 0.01). Therefore, Hypothesis H1a was verified. Model 3 added a moderator (migrant children’s identification with the college entrance examination policy). This moderator had a significant positive predictive effect on academic achievement expectations (*β* = 0.802, *p* < 0.01). Therefore, Hypothesis H2a was substantiated. Model 4 first conducted centralized processing of school class segregation and migrant children’s identification with the college entrance examination policy, and then built a product of the two terms. The product of the two terms had a positive predictive effect on academic achievement expectations (*β* = 0.148, *p* < 0.01). Meanwhile, the regression coefficient of the independent variable (school class segregation) to the dependent variable (interpersonal expectations) changed from −0.789 to −0.413, the absolute value of which decreased. This shows that migrant children’s identification with the college entrance examination policy might weaken the negative predictive effect of school class segregation on academic achievement expectations. Therefore, Hypothesis H3a was verified.

#### 4.2.2. Testing of the Moderating Effects of Policy Identification on the Relationship between School Class Segregation and Moral Performance Expectations

Table 4 shows the results of testing the moderating effects of migrant children’s identification with the college entrance examination policy on the relationship between school class segregation and moral performance expectations. The explanatory powers of the four-order model were 0.408, 0.565, 0.764, and 0.765, respectively, and all variables could explain 76.5% of the variance of the dependent variable. The F test showed that the explanatory powers were statistically significant.

On the basis of Model 1, Model 2 added an independent variable (school class segregation). This variable had a significant negative predictive effect on moral performance expectations (*β* = −0.784, *p* < 0.01). Therefore, Hypothesis H1b was verified. Model 3 adds a moderator (migrant children’s identification with the college entrance examination policy). This moderator had a significant positive predictive effect on moral performance expectations (*β* = 0.815, *p* < 0.01). Therefore, Hypothesis H2b was verified. Model 4 first conducted centralized processing of school class segregation and migrant children’s identification with the college entrance examination policy, and then built a product of the two terms. However, the positive predictive effect of the product of the two terms on moral performance expectations did not pass the significance test (*β* = 0.073, *p* > 0.05). Therefore, Hypothesis H3b was not verified.

#### 4.2.3. Testing of the Moderating Effects of Policy Identification on the Relationship between School Class Segregation and Interpersonal Expectations

Table 5 shows the results of testing the moderating effects of migrant children’s identification with the college entrance examination policy on the relationship between school class segregation and interpersonal expectations. The explanatory powers of the four-order model were 0.406, 0.601, 0.766, and 0.768, respectively, and all variables could explain 76.8% of the variance of the dependent variable. It was found through the F test that the explanatory powers were statistically significant.

On the basis of Model 1, Model 2 added an independent variable (school class segregation). This variable had a significant negative predictive effect on interpersonal expectations (*β* = −0.761, *p* < 0.01). Therefore, Hypothesis H1c was substantiated. Model 3 included a moderator (migrant children’s identification with the college entrance examination policy). This moderator had a significant positive predictive effect on interpersonal expectations (*β* = 0.847, *p* < 0.01). Therefore, Hypothesis H2c was verified. Model 4 first conducted centralized processing of school class segregation and migrant children’s identification with the college entrance examination policy, and then built a product of the two terms. The product of the two terms had a positive predictive effect on interpersonal expectations (*β* = 0.220, *p* < 0.01). Meanwhile, the regression coefficient of the independent variable (school class segregation) to the dependent variable (interpersonal expectations) changed from −0.761 to −0.614, the absolute value of which decreased. This shows that migrant children’s identification with the college entrance examination policy could weaken the negative predictive effect of school class segregation on interpersonal expectations. Therefore, Hypothesis H3c was verified.

#### 4.2.4. Testing of the Moderating Effects of Policy Identification on the Relationship between School Class Segregation and Social Achievement Expectations

Table 6 shows the results of testing the moderating effects of migrant children’s identification with the college entrance examination policy on the relationship between school class segregation and social achievement expectations. The explanatory powers of the four-order model were 0.369, 0.546, 0.713, and 0.713, respectively, and all variables could explain 71.3% of the variance of the dependent variable. As such, the F test showed that the explanatory powers were statistically significant.

Based on Model 1, Model 2 included an independent variable (school class segregation). This variable had a significant negative predictive effect on social achievement expectations (*β* = −0.801, *p* < 0.01). Therefore, Hypothesis H1d was verified. Model 3 added a moderator (migrant children’s identification with the college entrance examination policy). This moderator had a significant positive predictive effect on social achievement expectations (*β* = 0.715, *p* < 0.01). Therefore, Hypothesis H2d was substantiated. Model 4 first conducted centralized processing of school class segregation and migrant children’s identification with the college entrance examination policy, and then built a product of the two terms. However, the positive predictive effect of the product of the two terms on social achievement expectations did not pass the significance test (*β* = 0.067, *p* > 0.05). Therefore, Hypothesis H3d was not verified.

## 5. Discussion

Prior studies initially focused more on the relationship between school class composition and students’ academic achievement and final educational attainment [43,44]. Later, the relationship between students’ educational expectations and their final educational attainment started attracting increasing attention [45,46]. Scholars such as Wu and Huang [8] and Minello and Barban [6] believed that the higher the school class composition, the higher the educational expectations of migrant children. This is consistent with the research results in this paper, which show that school class segregation had significant negative impacts on migrant children’s academic achievement expectations, moral performance expectations, interpersonal expectations, and social achievement expectations.

School class segregation in Western countries stems from the self-selection and interaction of different class groups at the micro-level [15,16]. The phenomenon of school class segregation in China has certain unique characteristics, including the macro-level education policies and the differentiation in the quality of school education under policy changes. As such, the purpose of this study was to explore whether migrant children’s identification with the college entrance examination policy had moderating effects on the relationship between school class segregation and the educational expectations of migrant children. This study found that migrant children’s identification with the college entrance examination policy had significant positive effects on the four dimensions of educational expectations of migrant children. In other words, if migrant children support and agree with the college entrance examination policy for migrant children, then they are likely to develop good educational expectations.

Through regression analysis, this study verified the moderating effects of migrant children’s identification with the college entrance examination policy on the relationship between school class segregation and the educational expectations of migrant children. The results showed that migrant children’s identification with the college entrance examination policy weakened the negative effects of school class segregation on their academic achievement expectations and interpersonal expectations, but its effects on moral performance expectations and social achievement expectations were not verified. The college entrance examination policy for migrant children has opened up channels for migrant children to take the college entrance examination in the inflow cities. Even if school class segregation still exists, policy support makes migrant children feel accepted and recognized, which would help them develop good educational expectations.

The present study has the following contributions. First, this study enriches the research contents in this field and provides a Chinese perspective for the research on this issue. The issue of school class segregation in China largely involves China’s college entrance examination household registration system. This study considers that the reform of the college entrance examination policy for migrant children may have an impact on the formation of the educational expectations for migrant children. Therefore, this paper incorporates a variable (migrant children’s identification with the college entrance examination policy) into the regression model.

Second, this study takes into account the multidimensional nature of educational expectations. Migrant children are different from urban students of the same age. Due to the influence of the social environment and policy environment, social adaptability aspects such as morality and interpersonal relationships are also expected to be the focus of attention. In addition to considering the academic achievement expectations and social achievement expectations of migrant children, this study also accounts for their moral performance expectations and interpersonal expectations.

Third, this study also carries a great practical significance. The education issue of migrant children has always been a challenge facing the educational sector in China. China’s household registration policy is closely linked to its college entrance examination policy, which has exacerbated the issue of unfairness in education. The college entrance examination policy for migrant children aims to provide fair educational opportunities for migrant children. This study not only provides new evaluation support for assessing the implementation of the college entrance examination policy for migrant children but also offers theoretical support and policy recommendations for practical issues related to migrant children’s school selection in China.

### 5.1. Implications

Informed by the research findings of this study, this study makes the following proposals to mitigate the negative impacts of school class segregation on the educational expectations of migrant children.

First, the institutional mechanism involving the “unified urban–rural household registration” shall be established. The household registration system actually limits the speed and model in which migrant children can accumulate human capital, causing them to fall into a vicious cycle of “low educational expectations–low educational attainment–low human capital”. Currently, the college entrance examination systems in some provinces in China have removed the restriction on “whether the student has local household registration”. In essence, it is an institutional mechanism that helps to form a “unified urban–rural household registration”. This policy can not only promote migrant children to achieve true “urbanization” in terms of psychology, social roles, life, and study, but also provide them with the motivation of social mobility and promote them to establish good educational expectations.

Second, the cultural mechanism that involves “promoting learning through examinations” shall be developed. Through the ages, the ancient imperial examination system provided a channel for millions of poor students to rely on their efforts to gain social advancement. Since China resumed the college entrance examination in 1977, millions of students have taken the college entrance examination every year, and the number of students taking the college entrance examination continues to increase every year. As such, regardless of whether it is in ancient or modern times, selective examinations have a powerful function of “promoting learning through examinations”. It can motivate young people to learn, enhance the overall cultural literacy of all citizens, and pass on and carry forward outstanding culture. The provinces in China that took the lead in implementing the college entrance examination policy for migrant children have undoubtedly provided a cultural mechanism of “promoting learning through examinations” for migrant children on their way to study, encouraging them to establish good educational expectations.

Third, the compensation mechanism involving the “principle of justice” should be built. Rawls proposed in “A Theory of Justice” that society and the economy should “fit the interests of the least beneficiary”, that is, the difference principle [47]. The difference principle requires not only meeting the interests of everyone (i.e., the principle of efficiency), but also satisfying the interests of the weak under the condition of unequal efficiency and distribution of resources. The combination of the two is the “democratic equality system”. In the current social context, migrant children can be said to be one of the most disadvantaged groups among urban children. They should receive some compensation mechanism on the basis of fair opportunities. The provinces that took the lead in implementing the college entrance examination policy for migrant children have undoubtedly provided an effective compensation mechanism for fair education for migrant children.

### 5.2. Limitations and Future Research

Nonetheless, the present study has the following limitations. First, the sample size is insufficient. The current sample selection is based on principles of accessibility and convenience, which may result in a lack of comprehensiveness and representativeness of the research results. Future research may consider using national survey platforms such as the China Education Panel Survey (CEPS) and Chinese Family Panel Studies (CFPS) to embed some scales on the college entrance examination policy for migrant children and other aspects for a wider range of research. Second, the research hypotheses may not cover other important issues, such as the heterogeneity of educational expectations of migrant children at different ages. Future research is suggested to further improve the experimental design and take into account more possible influencing factors. Third, the research conclusions of this paper are solely based on quantitative data analysis, falling short of the support of qualitative data. Thus, research in the future may consider using interviews to triangulate with survey data to gain more solid and generalizable results.

## Figures and Tables

**Table 1:** Demographic characteristics of the sample.

Variables	*n* (%)	Distribution of Samples									
		ZJ	FJ	JS	SD	AH	JX	HB	HN	HB	HN
Sex											
Female	813 (45.9)										
Male	957 (54.1)										
Type of household mobility											
“Rural–urban” mobility	1405 (79.4)	218	125	160	156	98	133	129	103	118	165
“Town–town” mobility	365 (20.6)	65	40	33	30	39	38	32	33	34	21
School type											
Voluntary school (migrant children)	441 (24.9)	65	30	55	39	36	58	59	30	31	38
Local public school	1329 (75.1)	218	135	138	147	101	113	102	106	121	148
Grade											
Grade 8	532 (30.1)	83	27	30	46	34	66	101	38	54	53
Grade 9	618 (34.9)	115	23	69	82	53	105	60	20	35	56
Grade 10	312 (17.6)	47	57	51	25	22	0	0	43	31	36
Grade 11	308 (17.4)	38	58	43	33	28	0	0	35	32	41
Duration of residence											
1–2 years	201 (11.4)	24	22	27	17	12	34	26	6	16	17
3–4 years	864 (48.8)	145	41	75	109	64	113	108	52	74	83
5–6 years	500 (28.2)	96	67	60	45	39	15	21	58	41	58
≥7 years	205 (11.6)	18	35	31	15	22	9	6	20	21	28

N = 1770; ZJ = Zhe Jiang; FJ = Fu Jian; JS = Jiang Su; SD = Shan Dong; AH = An Hui; JX = Jiang Xi; HB = Hui Bei; HN = Hu Nan; HB = He Bei; HN = He Nan.

**Table 2:** Correlation analysis of values (n = 1770).

	(1)	(2)	(3)	(4)	(5)	(6)
(1) SCS	1					
(2) ICEEP	−0.593 **	1				
(3) AAE	−0.733 **	0.737 **	1			
(4) MPE	−0.734 **	0.679 **	0.721 **	1		
(5) IE	−0.755 **	0.628 **	0.894 **	0.826 **	1	
(6) SAE	−0.726 **	0.670 **	0.800 **	0.820 **	0.804 **	1

** *p* < 0.01; SCS= school class segregation; ICEEP = migrant children’s identification with the college entrance examination policy; AAE = academic achievement expectations; MPE = moral performance expectations; IE = interpersonal expectations; SAE = social achievement expectations.

**Table 3:** Moderating effect of ICEEP on the relationship between SCS and AAE.

Variables	Model 1		Model 2		Model 3		Model 4	
	Coefficients	SE	Coefficients	SE	Coefficients	SE	Coefficients	SE
(Constant)	4.340 **	0.339	5.312 **	0.303	1.794 **	0.315	1.776 **	0.317
Sex	−0.067	0.095	−0.026	0.081	0.152 *	0.060	0.155 *	0.060
Type of household mobility	0.153	0.110	0.091	0.093	−0.072	0.068	−0.074	0.068
Personnel structure	0.289 **	0.065	0.226 **	0.056	−0.056	0.044	−0.053	0.044
Duration of Residence	−0.556 **	0.065	−0.245 **	0.063	−0.107 *	0.046	−0.102	0.047
SCS			−0.789 **	0.077	−0.417 **	0.061	−0.413 **	0.061
ICEEP					0.802 **	0.052	0.774 **	0.052
SCS × ICEEP							0.148 **	0.070
ΔR^2^	0.367		0.555		0.761		0.767	
F	132.473 **		155.030 **		224.395 **		308.688 **	

* *p* < 0.05; ** *p* < 0.01; SCS= school class segregation; ICEEP = migrant children’s identification with the college entrance examination policy; AAE = academic achievement expectations.

**Table 4:** Moderating effect of ICEEP on the relationship between SCS and MPE.

Variables	Model 1		Model 2		Model 3		Model 4	
	Coefficients	SE	Coefficients	SE	Coefficients	SE	Coefficients	SE
(Constant)	4.826 **	0.347	5.790 **	0.314	1.794 **	0.315	1.776 **	0.317
Sex	−0.077	0.098	−0.010	0.084	0.152 *	0.060	0.155 *	0.060
Type of household mobility	0.052	0.112	−0.010	0.097	−0.072	0.068	−0.074	0.068
Personnel structure	0.298 **	0.067	0.236 **	0.058	−0.056	0.044	−0.053	0.044
Duration of Residence	−0.610 **	0.066	−0.301 **	0.065	−0.107 *	0.046	−0.102	0.047
SCS			−0.784 **	0.080	−0.417 **	0.061	−0.413 **	0.061
ICEEP					0.802 **	0.052	0.774 **	0.052
SCS × ICEEP							0.148 **	0.070
ΔR^2^	0.408		0.565		0.764		0.765	
F	136.610 **		157.322 **		222.056 **		206.913 **	

* *p* < 0.05; ** *p* < 0.01; SCS = school class segregation; ICEEP = migrant children’s identification with the college entrance examination policy; MPE = moral performance expectations; SE = standard error.

**Table 5:** Moderating effect of ICEEP on the relationship between SCS and IE.

Variables	Model 1		Model 2		Model 3		Model 4	
	Coefficients	SE	Coefficients	SE	Coefficients	SE	Coefficients	SE
(Constant)	4.995 **	0.404	6.245 **	0.349	1.794 **	0.315	1.776 **	0.317
Sex	−0.048	0.114	0.038	0.094	0.152 *	0.060	0.155 *	0.060
Type of household mobility	0.124	0.131	0.044	0.107	−0.072	0.068	−0.074	0.068
Personnel structure	0.375 **	0.078	0.294 **	0.064	−0.056	0.044	−0.053	0.044
Duration of Residence	−0.732 **	0.077	−0.332 **	0.072	−0.107 *	0.046	−0.102	0.047
SCS			−0.761 **	0.089	−0.417 **	0.061	−0.413 **	0.061
ICEEP					0.802 **	0.052	0.774 **	0.052
SCS × ICEEP							0.148 **	0.070
ΔR^2^	0.406		0.601		0.766		0.768	
F	138.099 **		168.978 **		223.670 **		208.827 **	

* *p* < 0.05; ** *p* < 0.01; SCS = school class segregation; ICEEP = migrant children’s identification with the college entrance examination policy; IE = interpersonal expectations; SE = standard error.

**Table 6:** Moderating effect of ICEEP on the relationship between SCS and IE.

Variables	Model 1		Model 2		Model 3		Model 4	
	Coefficients	SE	Coefficients	SE	Coefficients	SE	Coefficients	SE
(Constant)	4.716 **	0.343	5.702 **	0.307	1.794 **	0.315	1.776 **	0.317
Sex	−0.081	0.097	−0.012	0.082	0.152 *	0.060	0.155 *	0.060
Type of household mobility	0.057	0.111	−0.006	0.094	−0.072	0.068	−0.074	0.068
Personnel structure	0.268 **	0.066	0.204 **	0.057	−0.056	0.044	−0.053	0.044
Duration of Residence	−0.580 **	0.065	−0.264 **	0.063	−0.107 *	0.046	−0.102	0.047
SCS			−0.801 **	0.078	−0.417 **	0.061	−0.413 **	0.061
ICEEP					0.802 **	0.052	0.774 **	0.052
SCS × ICEEP							0.148 **	0.070
ΔR^2^	0.369		0.546		0.713		0.713	
F	132.682 **		155.383 **		197.110 **		184.979 **	

* *p* < 0.05; ** *p* < 0.01; SCS = school class segregation; ICEEP = migrant children’s identification with the college entrance examination policy; SAE = social achievement expectations; SE = standard error.

## Data Availability

The data presented in this study are available on request from the corresponding author. The data are not publicly available, due to ethical reasons.
